# Double-coated PLGA nanoparticles with hierarchical surface architecture for CD44-targeted siRNA delivery

**DOI:** 10.1007/s13346-026-02115-8

**Published:** 2026-05-08

**Authors:** Giuseppe Longobardi, Pini Shekhter, Claudia Conte, Ronit Satchi-Fainaro, Fabiana Quaglia

**Affiliations:** 1 Department of Pharmacy, University of Naples Federico II, 80131 Naples, Italy; 2 Department of Cancer Biology & Immunology, Gray Faculty of Medical and Health Sciences, Gray School of Medical Sciences, Tel Aviv University, 6997801 Tel Aviv, Israel; 3 Tel Aviv University Center for Nanoscience and Nanotechnology, Tel Aviv University, 6997801 Tel Aviv, Israel; 4 Sagol School of Neuroscience, Tel Aviv University, 6997801 Tel Aviv, Israel

**Keywords:** PLGA nanoparticles, Surface engineering, siRNA delivery, CD44-mediated targeting, 3D models

## Abstract

Efficient delivery of small interfering RNA (siRNA) remains a materials challenge because it requires nanocarriers that stabilize polyanionic cargo, support cellular interactions, and enable cytosolic delivery. Although poly(lactic-co-glycolic acid) (PLGA) nanoparticles (NPs) are used due to biocompatibility, biodegradability, and regulatory acceptance, siRNA delivery with PLGA requires interfacial engineering to meet these constraints. Here, a modular double-coated PLGA NP platform (dcNPs2.0) is developed and optimized for siRNA complexation, surface functionalization, and scalable manufacturing. The system comprises a PLGA core coated with a polyethyleneimine (PEI) interlayer to mediate siRNA binding, followed by a hyaluronic acid (HA) outer layer, which improves colloidal stability and promotes CD44-mediated uptake. Process optimization, including transition from batch nanoprecipitation to microfluidic fabrication, provides high yield, excellent reproducibility, narrow size distributions, and increased siRNA loading. X-ray photoelectron spectroscopy confirms hierarchical multilayer assembly. The optimized dcNPs2.0 formulation exhibited robust physicochemical stability during storage, in serum-containing media, and following lyophilization with appropriate cryoprotection. Functional evaluation of dcNPs2.0 demonstrated efficient HA-mediated cellular uptake and effective silencing following siRNA delivery in both two-dimensional monolayers and three-dimensional spheroids of MDA-MB-231 cells. Overall, this work establishes a scalable, rationally engineered PLGA nanoplatform that integrates extracellular targeting with intracellular delivery requirements for siRNA therapeutic applications.

**Graphical Abstract:**

Schematic representation of the development and evaluation of double-coated nanoparticles (dcNPs) for siRNA delivery. dcNPs were designed with a poly(lactide-co-glycolic acid) (PLGA) core, polyethyleneimine (PEI), siRNA, and a hyaluronan (HA) outer coating. The formulation process was optimized from conventional nanoprecipitation, which showed batch-to-batch variability, through process refinement to improve concentration conditions, siRNA loading, and reproducibility, and finally translated to a microfluidic platform (dcNPs 2.0), enabling highly monodisperse, reproducible, and scalable NP production. The optimized dcNPs2.0 were then biologically evaluated for CD44-mediated cellular uptake and intracellular siRNA release, leading to gene silencing in 2D and 3D cancer cell models. Created with BioRender.

**Figure:**
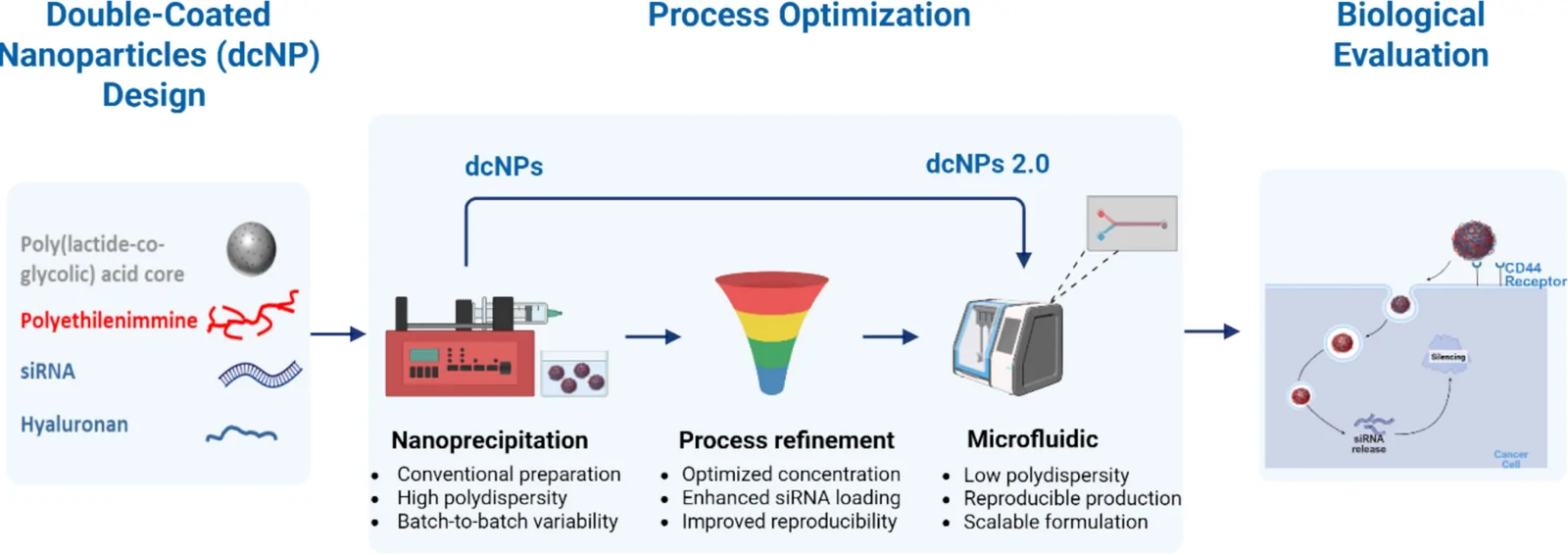

**Supplementary Information:**

The online version contains supplementary material available at https://doi.org/10.1007/s13346-026-02115-8.

## Introduction

Despite major clinical advances, efficient and selective small interfering RNA (siRNA) delivery remains a central materials challenge, with nanoparticle (NP) design playing a decisive role in overcoming extracellular and intracellular barriers [1–3]. Although polymeric NPs have been extensively explored for drug delivery [4–6], their application to siRNA delivery remains challenging, as it imposes more stringent and often conflicting interfacial design requirements [7]. In this context, although lipid NPs currently represent the clinical benchmark for RNA delivery, polymer-based NPs offer complementary advantages in terms of structural stability, formulation robustness, and manufacturing control, albeit at the cost of reduced intrinsic nucleic acid–membrane interactions [8].

Among biodegradable polymers, poly(lactide-co-glycolide) (PLGA) represents an attractive material owing to its regulatory acceptance and processability. However, in their native form, PLGA NPs are suboptimal for tumor targeting, as rapid immune recognition limits circulation time and effective accumulation at the target site [9]. These limitations become particularly critical for nucleic acid delivery, which requires stable complexation and protection of the hydrophilic cargo, as well as efficient cytosolic access to achieve biological activity.

Core–shell nanoconstructs, in which a hydrophilic shell surrounds a lipophilic polyester core, enable rational tailoring of NP interfaces to address specific therapeutic tasks and precisely control interactions with biological environments [10–15]. Within this framework, PEGylated PLGA NPs functionalized with targeting ligands represent the most widely adopted material design to modulate NP-biological interactions and biodistribution [9, 16]. However, such approaches typically rely on permanent chemical modification, limiting modularity and increasing synthetic complexity [17–25].

Biopolymer-based coatings represent a particularly attractive and modular surface-engineering strategy, as they avoid many of the limitations associated with direct chemical modification of polymeric materials. In chemically modified systems, covalent conjugation of ligands, hydrophilic chains, or targeting moieties typically requires multistep synthetic routes, often under harsh reaction conditions, which can compromise scalability and payload integrity.

Hyaluronic acid (HA) is a high-molecular-weight glycosaminoglycan and a key component of the extracellular matrix (ECM), widely employed in approved medicines and medical devices. Surface coating of NPs with HA enables multivalent interactions with the CD44 receptor, the primary HA receptor [26], which is frequently upregulated in several tumor types, including breast, colorectal, ovarian, prostate, and head and neck squamous cell carcinomas [27]. HA binds to the CD44 variant isoforms (CD44v), which are abundantly expressed on cancer cells and tumor-initiating cancer stem cells [28]. Beyond CD44, HA also interacts with CD44-like receptors, such as the HA-mediated motility receptor (HAMMR) and lymphatic vessel endothelial receptor-1 (LYVE-1), which are also overexpressed in tumor tissues [29]. Owing to its structural flexibility, HA can adopt multiple conformations to maximize receptor engagement, thereby promoting CD44 clustering [30, 31].

From a formulation perspective, HA coatings can be applied on PLGA NPs through mild and scalable processes, such as adsorption or layer-by-layer assembly, making them compatible with sensitive payloads and good manufacturing procedures (GMP) requirements. In addition to targeting, HA contributes to NP stability and stealth-like behavior through its highly hydrated structure. While PEGylation remains the gold standard for extending circulation time, emerging concerns about anti-PEG immunogenicity have sparked interest in HA as a safer alternative [7, 32]. Moreover, HA degradation by hyaluronidases, which are overexpressed in the tumor microenvironment, can be exploited to enable tumor-specific drug release, introducing an element of intrinsic stimuli-responsiveness [33].

We previously developed and extensively characterized double-coated NPs (dcNPs), which can be freeze-dried while retaining high stability in pharmaceutical vehicles and in the presence of plasma or fetal bovine serum. dcNPs selectively accumulate in CD44-overexpressing tumor cells, including A549 [34], HCT-116 [35], and MDA-MB-231 [36], as well as in cancer stem cells in different breast cancer models [37]. These dcNPs were shown to enable effective delivery of pDNA encoding L3 in HCT-116 cells, supporting their relevance for gene-based therapies. Building on this established platform and on the growing interest in siRNA-based strategies for solid tumors [38, 39], we extended the dcNP approach to siRNA delivery as a targeted nanotechnological strategy for CD44-overexpressing cancers.

In this study, we establish a robust and scalable methodology for the production of siRNA-loaded dcNPs, progressing from bench-scale fabrication to microfluidic manufacturing, evaluating their safety profile, and demonstrating their ability to silence luciferase and/or GFP in both two-dimensional (2D) and three-dimensional (3D) models of triple-negative breast cancer (TNBC).

## Results and discussion

### Optimization of the preparation protocol

**Fig. 1:**
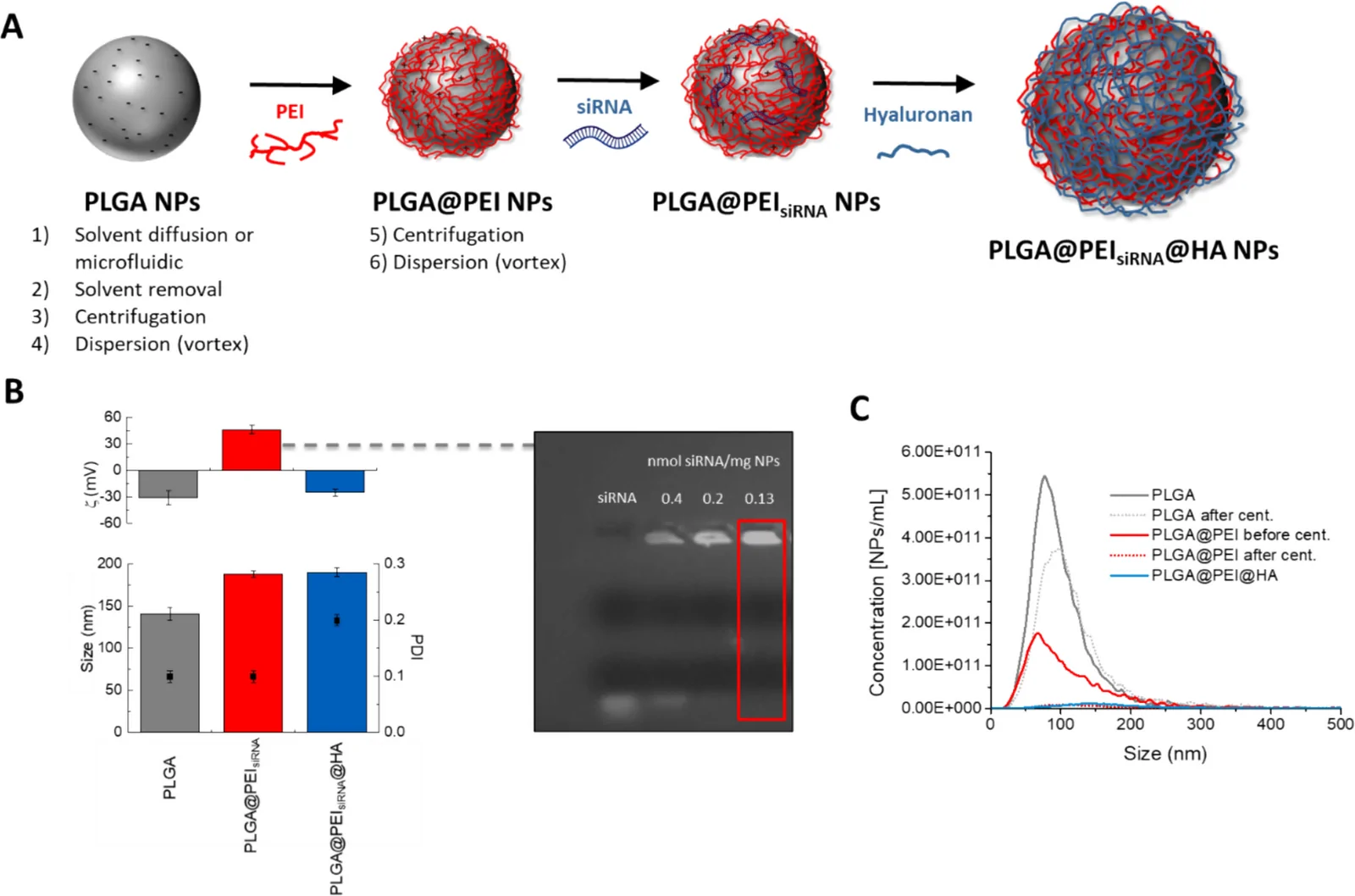
Overview of the dcNP layering procedure and its impact on NP properties. (**A**) Layering procedure to obtain dcNPs. (**B**) Evolution of D_H_, PDI, and ζ at each step of the layering procedure. Agarose gel electrophoresis showing the binding of siRNA_SC_ to PLGA@PEI NPs. (**C**) Evolution of NP concentration during the formulation process. Data represent the mean of three independent measurements from three different NP batches ± SD

dcNPs are obtained by a multistep procedure in which the PLGA core of NPs is coated with polyethyleneimine (PEI) (PLGA@PEI NPs), subsequently complexed with siRNA, and finally coated with HA (PLGA@PEI@HA NPs) (Fig. 1A). Multiple centrifugation-redispersion steps are required to apply the coatings, and these steps are considered critical, as they directly affect NP concentration. Importantly, the amount of NPs regulates the surface area available for PEI and HA adsorption via electrostatic interactions.

In a first attempt, we produced siRNA scramble (siRNA_SC_) -loaded dcNPs according to the procedure previously employed to load small drugs [34, 36, 40] or pDNA [35]. Each step was systematically monitored by measuring the average hydrodynamic diameter (D_H_), polydispersity index (PDI), and zeta potential (ζ) **(**Fig. 1B**)**. PLGA@PEI NPs became positively charged upon PEI addition (+ 46 ± 5 mV). Complete siRNA adsorption was achieved at a concentration of 0.13 nmol per mg of NPs, as demonstrated by the disappearance of the free siRNA band when monitored in an agarose gel electrophoresis. Notably, since the number of PEI protonated amine groups is in large excess to siRNA negative groups, the overall ζ remains positive upon siRNA complexation. Therefore, HA adsorption reversed the ζ, which became negative again (−25 ± 4 mV). Concomitantly, the initial size of PLGA NPs increased by approximately 40 nm, further supporting the formation of a PEI/HA multilayer incorporating siRNA.

Because the preparation protocol involves multiple centrifugation-dispersion steps, NP concentration is expected to decrease. Indeed, NP concentration directly determines the surface area available for PEI adsorption, siRNA complexation, and final HA coating, as well as the overall yield of the product. NP Tracking Analysis (NTA) measurements were collected after each step: (i) the PLGA core formation, (ii) the first washing step, (iii) after PEI coating, (iv) the subsequent washing step, and (v) the final formulation (Fig. 1C). Following these steps, NP concentration decreased by ~ 46-fold. This reduction corresponds to a proportional loss in total available surface area, as overall NP surface scales with particle number.

**Fig.2:**
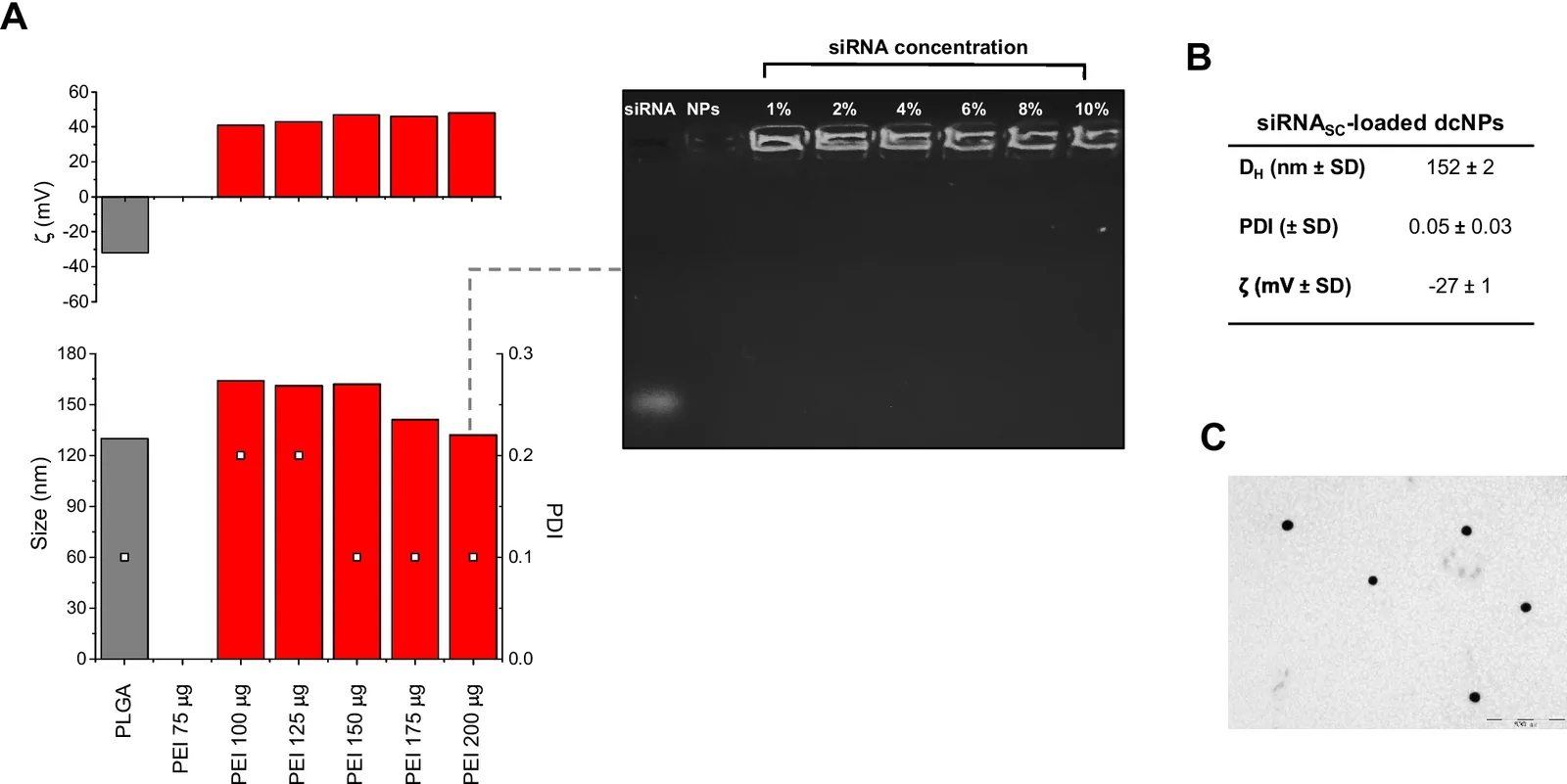
Optimization of dcNPs. (**A**) Evolution of PLGA NPs' colloidal properties at increasing concentrations of PEI. Agarose gel electrophoresis showing siRNA binding to PLGA@PEI (prepared at 200 μg of PEI). siRNA_SC_ ranges from 1 to 10% (mg siRNA/100 mg of NPs). (**B**) Properties of PLGA@PEI@HA NPs loaded with 4% siRNA_SC_. Data represent the mean of three independent measurements from three different NP batches ± SD. (**C**) TEM image of PLGA@PEI@HA NPs

Based on these findings, we optimized the preparation protocol to improve yield and robustness. Optimization focused on the PEI adsorption step by: (i) eliminating poloxamer 188 to enhance PEI adsorption, and (ii) refining the PEI/NP ratio to ensure complete surface coverage and eliminate intermediate centrifugation-redispersion steps. We therefore systematically evaluated the effect of increasing PEI concentrations on D_H_, PDI, and ζ of the PLGA core NPs (Fig. 2A). At 200 μg/mL of PEI (corresponding to a PEI/PLGA weight ratio of 0.08 w/w), no changes in the colloidal properties were observed, and free PEI was undetectable in the medium even at 2% of the total PLGA amount, indicating complete adsorption onto PLGA NPs.

Next, we investigated the effect of increasing siRNA loading to enhance therapeutic payload and maximize gene silencing potential. We were able to increase the concentration from 1% in the original dcNPs to 4% in the optimized formulation (Fig. 2A and B). In parallel, the HA concentration was increased to 1.5 mg/mL to further stabilize the NP architecture. The combined optimization of PEI, siRNA, and HA resulted in a substantial reduction in washing steps, thereby simplifying the formulation workflow. As a final step, a filtration step was introduced to remove aggregates. TEM confirmed the spherical morphology of the optimized NPs (Fig. 2 C).

The reduction or elimination of washing steps minimized siRNA loss, increased formulation yield from approximately 50% to 90%, and significantly improved process scalability, supporting future translational development. Importantly, minimizing washing steps also reduced batch-to-batch variability in NP recovery, thereby contributing to a more uniform and reproducible siRNA complexation onto the PEI layer and maintaining a more consistent NP/siRNA ratio.

Following formulation optimization, we addressed scalability and reproducibility, which are critical for transitioning from laboratory-scale production to clinical manufacturing. Initially, PLGA cores were produced by nanoprecipitation using a syringe pump. To improve process control, we transitioned to microfluidic production, enabling precise control of flow rates, reagent concentrations, and mixing dynamics. This approach yielded NPs with markedly improved monodispersity, achieving PDI values of less than 0.1 (Table 1). This reduction in PDI is particularly relevant, as size homogeneity directly impacts formulation reproducibility, siRNA release kinetics, and uniform cellular uptake across different cell populations.

**Table 1:** Composition and properties of dcNPs2.0

	D_H_ (nm ± SD)	PDI (± SD)	ζ (mV ± SD)	DLC (%)	DLE (%)	Yield (%)
dcNPs2.0-siRNA_SC_	158 ± 2	0.05 ± 0	−30 ± 1			99 ± 1
dcNPs2.0-siRNA_SC_ filtered 0.45μm	147 ± 2	0.04 ± 0	−35 ± 1	4%	99 ± 1	80 ± 4

By employing microfluidic techniques, we generated a more homogeneous and precisely defined PLGA core, which enabled consistent PEI adsorption and reduced batch-to-batch variability. This improvement minimized premature siRNA release, yielding a highly reproducible formulation. Importantly, stepwise physicochemical characterization demonstrated that siRNA complexation induced only a slight increase in D_H_ (approximately 10 nm) compared with PLGA@PEI NPs, while the ζ remained positive prior to HA coating. Subsequent HA deposition shifted the surface charge to negative values, supporting successful layer-by-layer assembly (Table S1).

Moreover, the low PDI improved scalability, making the system compatible with industrial-scale manufacturing requirements. Overall, the transition to microfluidics bridged the gap between bench-scale formulation and scalable production, advancing the dcNPs platform toward a robust and clinically relevant siRNA delivery system for CD44-overexpressing solid tumors. Hereafter, this final formulation is referred to as dcNPs2.0, while the siRNA payload denotes specific formulations.

### X-ray photoelectron spectroscopy surface and depth profile analysis

**Fig. 3:**
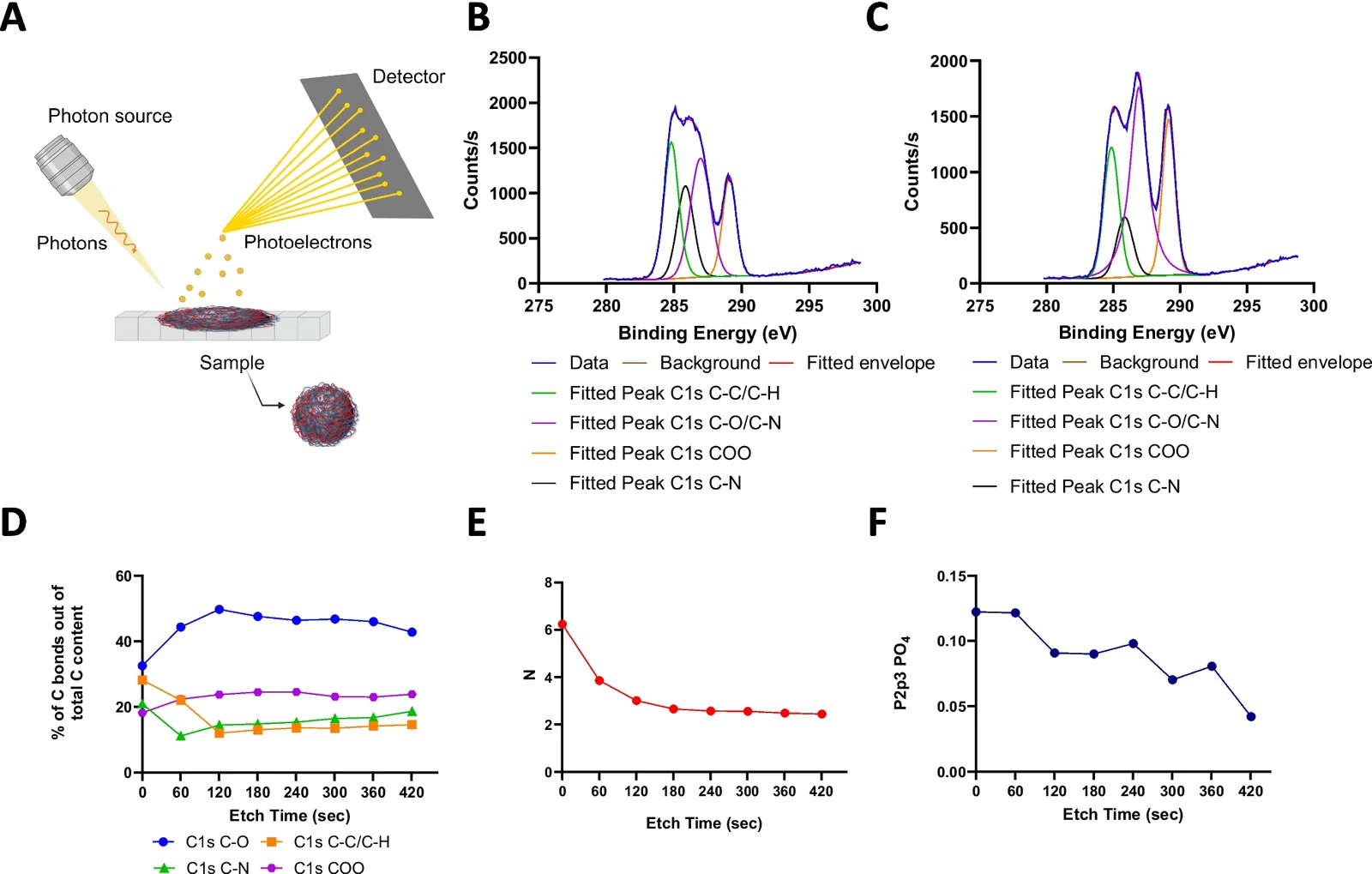
XPS depth profiling of dcNPs2.0-siRNA_SC_. (**A**) Schematic illustration of the XPS surface analysis and depth profiling workflow. XPS spectra were first acquired from the dcNP2.0 surface (no sputtering), followed by sequential Ar⁺ ion sputtering steps to probe subsurface composition from the exterior toward the interior. Created with BioRender. (**B**) High-resolution C1s spectrum acquired at the particle surface before sputtering (0 s). (**C**) High-resolution C1s spectrum acquired after 60 s of Ar⁺ ion sputtering during depth profiling. (**D**) Relative contribution of C1s components (C–C/C–H, C–O, C = O, and C–N) as a function of sputtering time (depth). (**E**) Atomic concentration of nitrogen (N, from the N 1 s region) as a function of sputtering time. (**F**) Atomic concentration of phosphorus (P, from the P 2p region; representative of siRNA phosphate groups) as a function of sputtering time

X-ray Photoelectron Spectroscopy (XPS) was employed to investigate the surface composition and layered architecture of the dcNPs2.0 (Fig. 3A). XPS is a surface-sensitive technique that provides elemental and chemical-state information from the outermost few nanometers of a material, making it particularly suitable for evaluating core–shell NP systems. In addition to standard surveys and high-resolution spectra, a depth profile analysis was performed using sequential sputtering, which enabled the assessment of the chemical composition from the particle surface toward the interior [41, 42].

High-resolution C1s spectra of the dcNP2.0 samples revealed four distinct carbon bonding environments (Fig. 3B-C), assigned to C–C, C–O, C = O, and C–N species. Of these, three bonds (C–C, C–O, and C = O) were also present in the PLGA NP core (SI Figure S1A), whereas the C–N bond was absent, consistent with the lack of nitrogen-containing functionalities in PLGA. Analysis of the relative contributions of these carbon species throughout the depth profile (Fig. 3D) demonstrated that the C–N signal is predominantly localized at the particle surface and decreases rapidly with increasing sputtering depth, indicating surface-enrichment of nitrogen-containing components.

This trend was further supported by the atomic concentration of nitrogen derived from the N1s spectra of the PEI-coated PLGA core (SI Figure S1B), which showed a marked decrease from the surface toward the particle interior (Fig. 3E). Similarly, the phosphorus signal, obtained from the P2p spectra of the same NPs after siRNA_SC_ adsorption (SI Figure S1C), exhibited a comparable depth-dependent reduction, consistent with surface-localized siRNA associated with the PEI layer (Fig. 3F). Taken together, these observations confirm that the PLGA core is effectively coated with the intended functional layers, with HA forming the outer interface while PEI and siRNA remain detectable within the near-surface region.

It should be noted that depth profiling was performed on an ensemble of radially oriented NP, rather than on a single, planar core–shell structure. As a result, in an ideal core–shell system, the outer shell can still contribute to the detected signal throughout the sputtering process. Consequently, although the data does not allow for the exclusion of partial interpenetration of surface components into the particle interior, the pronounced surface enrichment and rapid signal attenuation strongly suggest that any such penetration is limited. Overall, the XPS and depth profile analyses provide compelling evidence for the successful formation of a surface-functionalized, multilayered NP architecture.

### Storage, serum, and post-lyophilization stability of dcNPs2.0

**Fig. 4:**
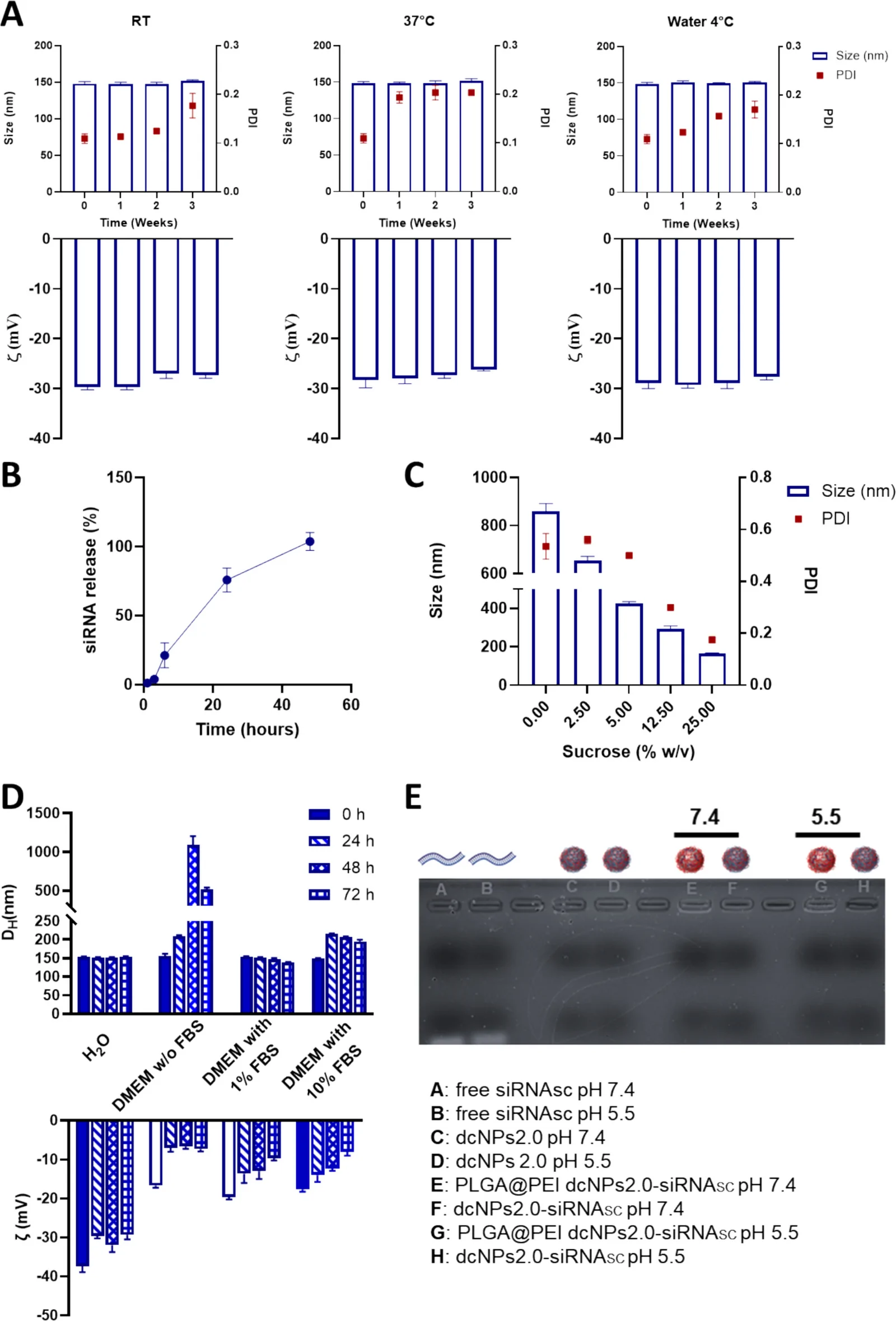
Physicochemical stability and siRNA retention of dcNPs2.0-siRNA_SC_. (**A**) Stability of dcNPs2.0-siRNA_SC_ evaluated at RT, 37 °C, and 4 °C over three weeks, showing preserved NP integrity under all tested storage conditions. (**B**) siRNA release profile of dcNPs2.0-siRNA_SC_ under physiological conditions at 37 °C. (**C**) Lyophilization studies assessing dcNPs2.0-siRNA_SC_ stability after freeze-drying in the presence of increasing sucrose concentrations (w/v%), demonstrating the protective effect of sucrose during lyophilization. (**D**) Stability in cell culture conditions, evaluated in media without serum and supplemented with 1% or 10% FBS, showing maintained dcNPs2.0-siRNA_SC_ integrity under biologically relevant conditions. (**E**) pH-dependent stability of siRNA complexed within dcNPs2.0 at pH 7.4 and pH 5.5, indicating stable siRNA association under both physiological and endosomal-like acidic conditions. Data are presented as mean ± SD, *n* = 3 independent experiments

The physicochemical stability of the dcNPs2.0-siRNA_SC_ formulation was comprehensively evaluated under a range of storage and biologically relevant conditions to ensure robustness throughout handling and application. First, NP stability was assessed at room temperature (RT), 4 °C, and 37 °C, showing overall preservation of colloidal properties over time (Fig. 4A). Notably, incubation at 37 °C led to a modest increase in PDI, consistent with mild thermally induced broadening of the size distribution. At the same time, the particles remained colloidally stable, with no evidence of major destabilization.

To evaluate siRNA release kinetics, dcNPs2.0-siRNA_SC_ were incubated under physiological conditions at 37 °C, where a gradual and sustained release profile was observed, reaching approximately 100% siRNA release within 48 h (Fig. 4B). This release behavior is consistent with the formation of a stable siRNA complex, followed by progressive release under physiological conditions.

To further assess formulation robustness and handling, lyophilization studies were conducted using increasing concentrations of sucrose (w/v%) as a cryoprotectant. The results demonstrated that sucrose effectively preserves NP integrity upon freeze-drying and subsequent reconstitution (Fig. 4 C), supporting the suitability of the formulation for long-term storage and transport.

We next valued colloidal stability in cell-relevant environments by incubating dcNPs2.0-siRNA_SC_ in culture medium supplemented with 1% or 10% Fetal Bovine Serum (FBS), as well as under serum-free conditions. The formulation remained stable for up to 72 h in the presence of 1% and 10% FBS, indicating strong compatibility with protein-containing media (Fig. 4D). In contrast, under serum-free conditions, NP showed pronounced aggregation after ~ 24 h, suggesting that serum proteins contribute to colloidal stabilization, likely through the formation of a stabilizing protein corona and/or screening of interparticle attractive interactions.

Finally, to assess siRNA retention under environments mimicking physiological and intracellular conditions, dcNPs2.0-siRNA_SC_ were incubated at pH 7.4 and pH 5.5. No detectable siRNA release was observed after 1 h at either pH, confirming stable siRNA complexation within the dcNPs2.0 and supporting protection against premature release during early trafficking (Fig. 4E).

Collectively, these results demonstrate that dcNPs2.0 exhibit high physicochemical stability across storage and biologically relevant conditions, controlled siRNA release, and robust performance in serum-containing biological media, supporting their suitability as a platform for siRNA delivery.

### Biological evaluation in 2D cell model

**Fig. 5:**
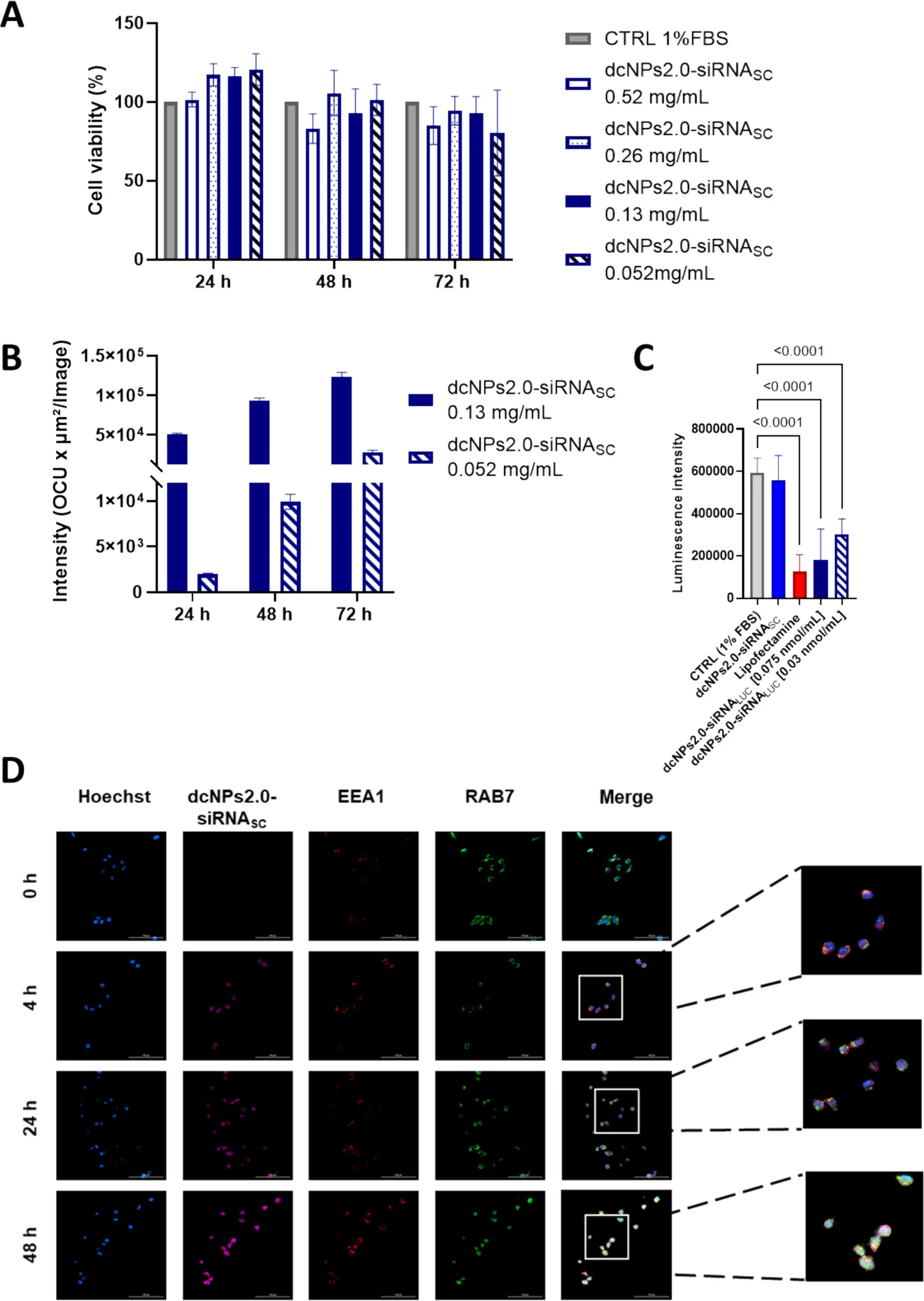
Biocompatibility, uptake, gene silencing, and endosomal trafficking of dcNPs2.0-siRNA in 2D TNBC cell models. (**A**) MDA-MB-231 viability after incubation with increasing concentrations of dcNPs2.0-siRNA_SC_ for up to 72 h, showing no detectable cytotoxicity. (**B**) Quantification of cellular uptake of rhodamine-labeled dcNPs2.0-siRNA_SC_ at two concentrations (0.13 and 0.052 mg/mL), monitored over 72 h by fluorescence readout, indicating efficient concentration-dependent internalization. (**C**) Luciferase knockdown in MDA-MB-231 cells stably expressing luciferase after treatment with dcNPs2.0-siRNA_LUC_ at 0.075 and 0.03 nmol/mL, demonstrating functional siRNA delivery and efficacy. dcNPs2.0-siRNA_SC_ were used as a negative control (0.075 nmol/mL) and Lipofectamine (0.075 nmol/mL) was used as a positive control. (**D**) Representative confocal images of dcNPs2.0-siRNA_SC_ intracellular localization relative to endosomal compartments at 4 h, 24 h, and 48 h: minimal colocalization with early endosomes at 4 h, more diffuse intracellular distribution at 24 h, and increased association with late endosomes at 48 h. Nuclei (blue), dcNPs2.0 (pink), EEA1 (red), RAB7 (green), and merged channels are shown. Insets indicate regions of interest; merged views are shown. Scale bar: 100 µm. Data are presented as mean ± SD of 3 independent experiments

To comprehensively evaluate the dcNPs2.0 for siRNA delivery in vitro, we first assessed its biocompatibility by incubating MDA-MB-231 cells with increasing concentrations of dcNPs2.0-siRNA_SC_ for up to 72 h. No detectable cytotoxicity was observed at any tested dose or exposure time, confirming that the NPs are well tolerated in this cellular model (Fig. 5A). This favorable safety profile, together with the previously demonstrated physicochemical stability across different media and pH conditions, supports the suitability of the formulation for intracellular delivery applications.

Cellular internalization was then investigated in a 2D model exploiting the high CD44 expression in TNBC cells, as confirmed by flow cytometry (SI Figure S2). To visualize uptake, dcNPs2.0 were fluorescently labeled by incorporating PLGA–Rhodamine. Quantitative fluorescence analysis revealed efficient and concentration-dependent internalization of NPs over 72 h, consistent with HA-mediated targeting of CD44 (Fig. 5B and SI Figure S3). Control experiments confirmed that incorporation of PLGA-Rhodamine did not alter the colloidal properties of NPs (Table S2).

The functional delivery of siRNA was subsequently evaluated using luciferase-targeting siRNA (siRNA_LUC_) in MDA-MB-231 cells stably expressing luciferase. Treatment with dcNPs2.0-siRNA_LUC_ resulted in a significant reduction in luciferase expression, demonstrating effective intracellular release and biological activity of the delivered siRNA (Fig. 5C). To correlate these functional outcomes with intracellular processing, endosomal trafficking was examined at 0, 4, 24, and 48 h post-incubation (Fig. 5D). At the early time point (4 h), dcNPs2.0 were readily internalized and showed minimal colocalization with early endosomal markers (early endosome antigen 1, EEA1), suggesting that CD44-mediated uptake may involve non-classical endocytic pathways and/or rapid transit through early endosomes. At 24 h, the NP signal appeared more diffusely distributed throughout the cytoplasm, with limited overlap with endosomal compartments, consistent with partial endosomal escape, likely facilitated by the proton sponge effect of the PEI interlayer[43, 44]. At 48 h, increased colocalization with the late endosomal marker (Ras-related protein Rab-7, RAB7) was observed, indicating that a fraction of internalized NPs undergoes delayed trafficking toward late endosomes, reflecting incomplete escape or endolysosomal recycling over time [45, 46]. Importantly, even at this later stage, a substantial proportion of the NP signal remained spatially separated from endosomal compartments, in agreement with the sustained gene silencing observed, and confirming that the nanosystem enables sufficient cytosolic release of siRNA to achieve effective target knockdown.

### Biological evaluation in 3D cell model

To further assess the performance of dcNPs2.0 in a more physiologically relevant setting, we extended our investigation to a 3D tumor model. Tumor spheroids generated from MDA-MB-231 cells were used, as they better recapitulate the architecture and microenvironment of solid tumors compared to conventional 2D cultures [47]. In particular, 3D spheroids reproduce key features such as cellular heterogeneity, cell–cell interactions, and diffusion barriers, which are known to significantly affect NP penetration, distribution, and therapeutic efficacy in vivo [48].

**Fig. 6:**
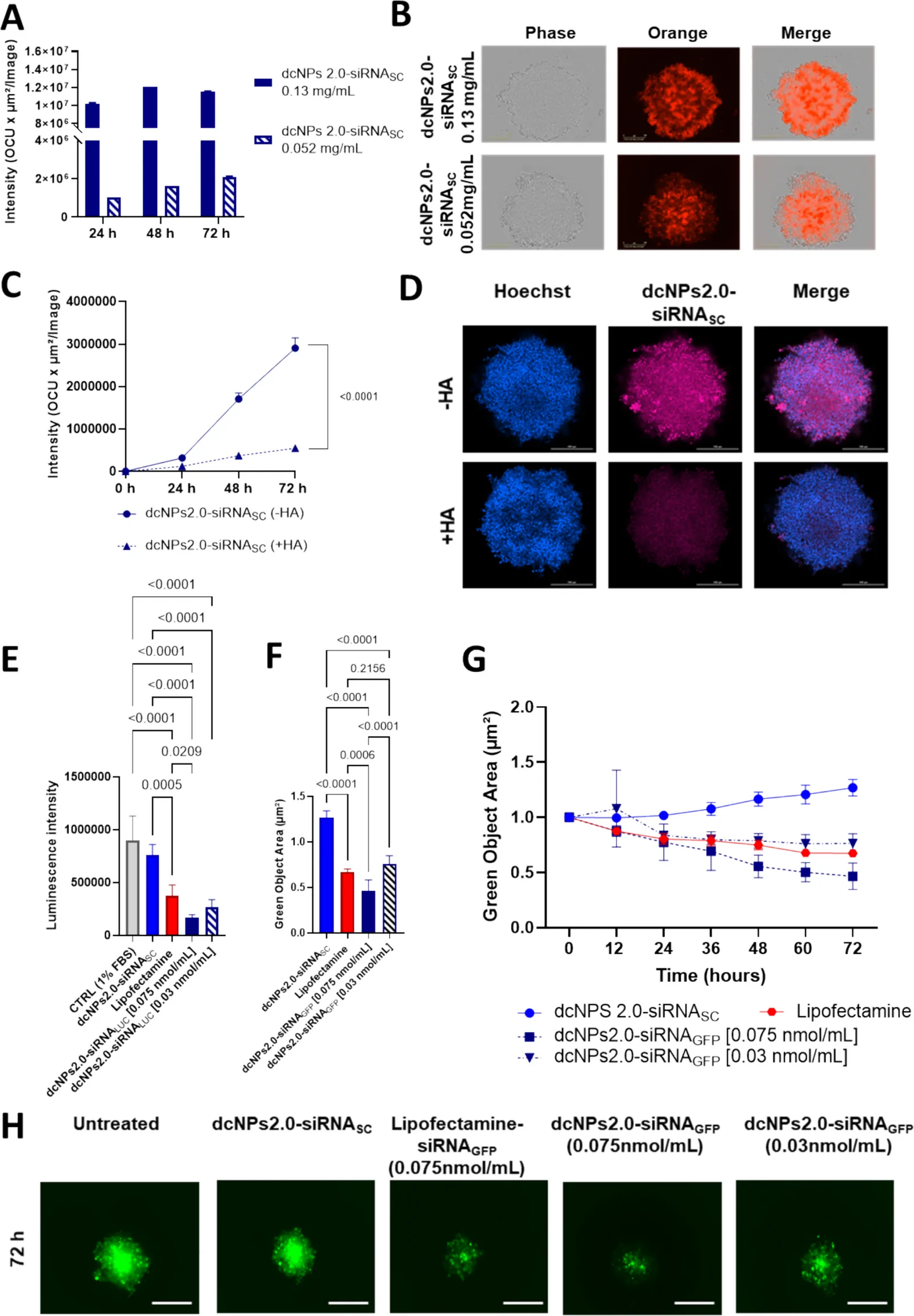
Uptake and gene silencing of dcNPs2.0-siRNA in 3D TNBC spheroid models. (**A**) Time-dependent internalization of PLGA–rhodamine–labeled dcNPs2.0-siRNA_SC_ in MDA-MB-231 spheroids at two dcNPs2.0 concentrations (0.13 and 0.052 mg/mL), monitored over 72 h by fluorescence readout. (**B**) Representative fluorescence images of spheroids after 72 h incubation with dcNPs2.0-siRNA_SC_ at 0.13 mg/mL (top) and 0.052 mg/mL (bottom). Scale bar: 400 µm. (**C**) Competitive uptake assay performed by pre-incubating spheroids with free HA (10 mg/mL) to saturate CD44, followed by treatment with dcNPs2.0-siRNA_SC_ (0.13 mg/mL) for 72 h; fluorescence signal quantification over time. (**D**) Representative confocal images after 72 h showing dcNPs2.0 localization within spheroids without (top) or with (bottom) HA pretreatment. Nuclei (blue), dcNPs2.0 (pink), and merged channels are shown. Scale bar: 200 µm. (**E**) Luciferase knockdown in MDA-MB-231 spheroids stably expressing luciferase following treatment with dcNPs2.0-siRNA_LUC_ at 0.075 and 0.03 nmol/mL for 72 h, demonstrating functional siRNA delivery and gene silencing. (**F**) GFP knockdown in MDA-MB-231 spheroids stably expressing GFP following treatment with dcNPs2.0-siRNA_GFP_ for 72 h. (**G**) Kinetics of GFP fluorescence decrease in GFP-expressing spheroids from 0 to 72 h post-treatment. (**H**) Representative fluorescence images after 72 h comparing untreated spheroids, spheroids treated with dcNPs2.0siRNA_SC_ (negative control), Lipofectamine-siRNA_GFP_ at 0.075 nmol/mL (positive control), and dcNPs2.0-siRNA_GFP_ at 0.075 and 0.03 nmol/mL to show dose response. Scale bar: 400 µm. Data are presented as mean ± SD, *n *= 3 independent experiments

We first evaluated the ability of the dcNPs2.0 to penetrate and be internalized within the spheroids, with a specific focus on CD44-mediated uptake. For this purpose, rhodamine-labeled PLGA dcNPs2.0 prepared using a PLGA/PLGA–Rhodamine blend were employed for fluorescent tracking, as previously performed in the 2D model. Over a 72 h incubation period, dcNP2.0-siRNA_SC_ efficiently penetrated the outer layers of the spheroids and were internalized by tumor cells in a concentration-dependent manner, consistent with the uptake trends observed in 2D cultures (Fig. 6A-B). Importantly, the 3D spheroid architecture imposed additional barriers, including a dense extracellular matrix and tightly packed cellular organization, which more stringently challenge NP transport and internalization [49].

To specifically isolate the contribution of CD44-mediated internalization, a competitive uptake assay was performed. Spheroids were pretreated with free HA to saturate CD44 receptors before exposure to rhodamine-labeled dcNP2.0-siRNA_SC_, while untreated spheroids served as controls. Pretreatment with free HA resulted in a marked reduction in NP internalization, as evidenced by a significant decrease in rhodamine fluorescence intensity (Fig. 6C), confirming the involvement of CD44 in NP transport. Confocal microscopy further supported these findings, revealing reduced NP accumulation within the spheroid core upon CD44 blockade (Fig. 6D).

Gene silencing efficiency was subsequently evaluated using siRNA_LUC_ as a model cargo. The NPs retained their structural integrity within the 3D spheroid environment and induced a significant reduction in bioluminescence, reflecting the decrease in luciferase expression, demonstrating effective siRNA delivery and functional gene silencing throughout the spheroids (Fig. 6E). To further visualize gene silencing at the functional level, MDA-MB-231 spheroids stably expressing GFP were treated with dcNPs2.0 loaded with GFP-targeting siRNA (siRNA_GFP_). Consistent with the luciferase data, a pronounced and time-dependent decrease in GFP fluorescence was observed within the spheroids, confirming efficient silencing of the fluorescent reporter protein (Fig. 6F–H).

From a materials design perspective, the performance observed in both 2D and 3D models highlights the synergistic role of the double-coating architecture, in which the outer HA layer promotes selective cellular interactions and penetration within tissue-like environments, while the underlying PEI interlayer supports intracellular processing and the functional release of siRNA. This hierarchical organization enables the decoupling of extracellular targeting from intracellular delivery requirements, a feature that is difficult to achieve with single-layer or covalently functionalized systems.

Overall, the ability of the NP system to penetrate 3D tumor spheroids, undergo CD44-mediated internalization, and induce gene silencing underscores its potential to overcome the physical and biological barriers characteristic of solid tumors. Together, these results highlight the promise of this nanoplatform for therapeutic siRNA delivery in complex, tissue-like environments expressing CD44, and support its further evaluation in advanced preclinical models.

## Conclusions

In this work, we developed and systematically optimized a modular, double-coated PLGA-based nanoplatform (called dcNPs2.0) specifically engineered for efficient siRNA complexation and delivery. Process optimization, including the transition to microfluidic fabrication and rational control of surface composition, resulted in high formulation yield, excellent colloidal stability, and reproducible NP properties. The hierarchical PEI/HA surface architecture enabled CD44-mediated cellular uptake, controlled intracellular processing, and effective gene silencing in both 2D and 3D models of TNBC, demonstrating functional siRNA delivery under increasingly complex biological conditions. Beyond siRNA delivery, the modular design of dcNPs2.0 provides a versatile materials platform that can be readily adapted to incorporate additional therapeutic payloads within the PLGA core. Overall, this work establishes a scalable and rationally engineered nanoplatform with strong potential for further development into combination and multimodal strategies for treating solid tumors.

## Experimental section

### Materials

PLGA (Resomer RG 502H, 50:50 with an inherent viscosity of 0.16–0.24 dL g^−1^), Agarose, Ethidium bromide (EtBr), and Tris–acetate–EDTA (TAE) buffer were obtained from Merck. PLGA-Rhodamine B (LG 50:50 Rhodamine B endcap Mn 10,000–30,000 Da) was obtained from PolySciTech (Microtech, Italy). Hyaluronan (HA, MW < 10 kDa) was a kind gift of Magaldi Life S.r.l. (Italy). PEI (MW ∼ 25 kDa branched) and Poloxamer 188 (Pluronic® F68) were purchased from Sigma-Aldrich (Merck, Italy). Silencer™ Firefly Luciferase (GL2 + GL3) siRNA (siRNA_Luc_) and Silencer™ Select Negative Control No. 1 siRNA (siRNA_sc_) were purchased from Invitrogen (Thermo Fisher Scientific, Italy), Silencer GFP™ siRNA (siRNA_GFP_), Lipofectamine 2000 Reagent, and the Quant-iT™ RiboGreen RNA Assay Kit were purchased from Rhenium (Israel). Dulbecco’s modified Eagle’s medium (DMEM), L-glutamine, and FBS were purchased from Gibco Thermo Fisher (Waltham, MA, USA). Penicillin–Streptomycin solution and Trypsin-Ethylenediaminetetraacetic Acid (EDTA) were purchased from Capricorn Scientific (Ebsdorfergrund, Germany). Paraformaldehyde (PFA) 4% solution was purchased from Thermo Scientific (Waltham, MA, USA). Dulbecco’s Phosphate Buffered Saline (PBS) was purchased from Sartorius (Goettingen, Germany). RAB7A Polyclonal Antibody (catalog no. 55469–1-AP-150UL) and EEA1 Monoclonal antibody (catalog no. 68065–1-IG-150UL) were purchased from Biotest (Israel). Goat Anti-Mouse IgG H&L (Alexa Fluor® 647) (catalog no. AB150115) and Goat Anti-Rabbit IgG H&L (Alexa Fluor® 488) (catalog no. AB150077) were purchased from Abcam (UK). PerCP anti-mouse/human CD44 Antibody (catalog no. 103035) was purchased from Enco Ltd (Israel). Isotype Control Antibody, rat IgG2b (catalog no. 130–102–658) was purchased from Y.A. Almog Diagnostic & Medical Equipment Ltd (Israel). Hoechst 33,342 and ProLong® Gold mounting were purchased from Invitrogen (Carlsbad, California, USA). Cell proliferation kit II (XTT), Sucrose, and TWEEN® 80 were purchased from Sigma-Aldrich (Rehovot, Israel). Luciferase Assay Kit (Promega) was purchased from IM Beit HaEmek (IMBH) (Israel). Dialysis maxi-GeBaFlex tubes (3.5 kDa Molecular weight cut-off) were purchased from Gene Bio-Application Ltd. (Yavne, Israel).

All buffers and solutions were prepared using analytical-grade reagents and ultrapure, RNase-free water.

### NPs preparation and characterization

dcNPs were prepared by a layer-by-layer deposition method according to our previous protocol [36]. PLGA cores were prepared by solvent diffusion, wherein 2 mL of 5 mg/mL PLGA in acetone were added dropwise (flow rate of the syringe pump at 1.5 mL/min) to an aqueous phase (4 mL water with 0.1% Poloxamer 188) under magnetic stirring. Following solvent removal under reduced pressure at (RT), the sample was split into four Eppendorf^®^ tubes and then centrifuged (8000 g, 15 min). After discarding the supernatant, the pellet was dispersed in 1 mL of water by gentle vortexing. For the coating procedure, 125 μL of a PEI solution (1 mg/mL) was added in each tube. The sample was centrifuged again (2800 g, 10 min), and the pellet was redispersed in 1 mL of water. siRNA (10 nmol) and then 100 μL of HA (1 mg/mL in water) were sequentially added. We set a 15-min interval between each step.

In dcNPs2.0, the PLGA cores were prepared using microfluidics in a NanoAssembler^®^ (Precision NanoSystems Inc., Vancouver, Canada) equipped with a BenchTop Cartridge (NIT0004), with the organic/aqueous phase ratio set at 1:2 and the total flow rate of 12 mL/min. Following microfluidic preparation, the PLGA core NPs were coated with PEI (200 μg/mL) and, after 15 min, complexed with siRNA. Finally, the NPs were coated with HA (1.5 mg/mL). The final formulation was filtered through a 0.45 μm RC filter.

A fluorescent variant of dcNPs2.0 was produced according to the procedures described above using a PLGA/PLGA-Rhodamine mixture (20:1).

The yield of the NP production process was evaluated by weighing the solid residue after freeze-drying an aliquot of NP dispersion. The yield is expressed as the ratio of the actual NP weight to the theoretical polymer or polymer + drug weight × 100 ± standard deviation (*n *= 3).

The morphology of the NPs was evaluated using transmission electron microscopy (TEM) (CM 12 Philips, Eindhoven, The Netherlands) after staining samples with a 2% w/v phosphotungstic acid solution (Phenom Prox).

XPS measurements were performed using a Thermo Scientific™ ESCALAB™ QXi, with an Al Kα source. Spot diameter of 200 μm was used with a pass energy of 20 eV. Dual-beam charge compensation was utilized. Depth profiling was performed using 500 Ar^+^ clusters with an energy of 4 keV, with 60-s steps to allow gentle removal of material without damaging organic bonds.

NP D_H_, PDI, and ζ were determined using dynamic light scattering (DLS) with a Zetasizer Nano ZS (Malvern Instruments Ltd.) and Möbius instrument (Wyatt Technology Corporation, Santa Barbara, CA, USA). The concentration of dcNPs was evaluated using a NanoSight Pro (Malvern Instruments Ltd.). Results are reported as the mean of three separate measurements on three different batches ± standard deviation (*n* = 3).

### siRNA complexation and quantification

The adsorption of siRNA onto the PEI layer of NPs was qualitatively confirmed by agarose gel retardation assays. Increasing amounts of siRNA were added to a fixed amount of NPs and loaded onto a 2% (w/v) agarose gel in TAE buffer, in the presence of EtBr. Electrophoresis was performed for 45 min at 60 V. Following separation, siRNA bands were visualized using a UV transilluminator.

The drug loading capacity (DLC) and drug loading efficiency (DLE) of siRNA were quantified indirectly using the Quant-iT™ RiboGreen RNA Assay Kit. Briefly, 4.2 mg of siRNA-loaded dcNPs were centrifuged at 30,000 rpm for 30 min at 4 °C. The supernatant was collected, and the amount of unbound siRNA was quantified according to the manufacturer’s protocol. DLC (wt%) and DLE (%) were calculated using the following equations:

$$DLC \left(\%\right)= \frac{initial \;mass \;of\; the\; siRNA-mass\; of\; siRNA\; in\; the\; supernetant}{NPs \;weight} x 100$$

$$DLE \left(\%\right)= \frac{mass \;of\; siRNA\;in \;NPs}{total \;mass \;of \;siRNA \;used\; in\; the\; formulation } x 100$$

### siRNA release profile

siRNA-loaded dcNPs2.0 were aliquoted into semipermeable dialysis tubes (1 mL) and dialyzed against DMEM supplemented with 1% FBS at 37 °C under gentle agitation. At predetermined time points (0, 3, 6, 24, and 48 h), aliquots of the NP suspension were collected and centrifuged at 30,000 rpm for 30 min at 4 °C. The supernatant was recovered, and the amount of released (unbound) siRNA was quantified using the Quant-iT™ RiboGreen RNA Assay Kit, as described above. Unloaded NPs subjected to the same procedure were used as blanks to correct for any background signal or potential interference from the NP components.

### dcNPs2.0 stability in biological simulated media

The colloidal stability of dcNPs2.0 was evaluated in water under different storing conditions (RT, 37 °C, and 4 °C) over a period of 3 weeks, with measurements of their colloidal properties (D_H_, PDI, ζ) by DLS. The stability under cell culture conditions was evaluated in DMEM supplemented with 2 mM L-glutamine and 50 U/mL penicillin–streptomycin, containing 0%, 1%, and 10% FBS over a 72 h period, corresponding to the duration of the biological experiments. To evaluate the stability of siRNA adsorbed onto NPs and its pH-responsive release, siRNA-loaded dcNPs2.0, uncoated and coated with HA, were incubated at pH 7.4 (physiological conditions) and pH 5.0 (endosomal acidic conditions). siRNA stability was qualitatively assessed by agarose gel retardation assays as described above.

### Lyophilization study

Freshly prepared NP suspensions were supplemented with sucrose at varying concentrations (2.5, 5, 12.5, and 25% w/v). The samples were rapidly frozen in liquid nitrogen and subsequently lyophilized for 24 h. After lyophilization, the samples were reconstituted in water, and their colloidal properties (D_H_, PDI, ζ) were assessed using DLS.

### Cell culture

MDA-MB-231 cells (American Type Culture Collection, ATCC, Manassas, Virginia) were cultured in DMEM supplemented with 10% FBS, 2 mM L-glutamine and 50 U/mL penicillin–streptomycin, and maintained in a humidified atmosphere with 5% CO_2_ at 37 °C.

### Flow cytometry

For flow cytometry analysis of CD44 expression, 6 × 10^4^ MDA-MB231 cells were seeded on 6-well plates. After 24 h, the cells were detached using PBS supplemented with 5 mM EDTA, 1% FBS, and 0.1% sodium azide (FACS buffer). Cells were washed once with FACS buffer before surface staining. Cells were stained for CD44 for 20 min at 4 °C. The corresponding isotype controls were used as negative staining controls.

### Biological evaluation in a 2D MDA-MB-231 model

For uptake and biocompatibility studies, cells were seeded at a density of 2.5 × 10^3^ cells per well in a 96-well plate. After 24 h of incubation, the medium was gently removed by vacuum and replaced by fresh medium (1% FBS) containing treatments as detailed below.

The uptake of siRNA_SC_-loaded dcNP2.0 was monitored over 72 h by measuring rhodamine fluorescence with an Incucyte^®^ SX5 (Sartorius) using an orange filter.

Cell viability after 24, 48 and 72 h of incubation with dcNPs2.0 was assessed using the XTT assay (Sigma-Aldrich), measuring absorbance at 450 and 630 nm (BioTek Cytation 105, Agilent Technologies, Inc).

For the silencing assay, MDA-MB-231 cells transfected with the luciferase gene (MDA-MB-231-Luc) were seeded and incubated for 24 h, as previously described. After incubation, the medium was removed by vacuum, and fresh medium containing siRNA_LUC_ dcNPs2.0 was added. The cells were incubated for up to 72 h, after which luciferase silencing was evaluated using the Luciferase Assay Kit (Promega). Lipofectamine was used as a positive control. The luminescence was quantified using a BioTek Cytation 105 plate reader, providing a measure of gene silencing efficiency.

### Endosomal trafficking

To evaluate intracellular localization and endosomal trafficking, cells were seeded as described above in black, clear-bottom 96-well plates. After 24 h of incubation, the culture medium was gently removed by vacuum and replaced with fresh medium containing 1% FBS and dcNPs2.0-siRNA_sc_, prepared using a PLGA/PLGA–Rhodamine mixture as described above.

At predetermined time points (0, 4, 24, and 48 h), cells were fixed with 4% (w/v) PFA for 20 min at RT and washed with PBS. Cells were then permeabilized for 10 min at RT using a permeabilization buffer composed of 0.3% (v/v) Triton X-100 in PBS, and then washed 3 times with PBS. Then, cells were blocked for 2 h at RT with a blocking buffer containing 0.1% (w/v) bovine serum albumin (BSA), 10% (v/v) normal goat serum, and 0.3% (v/v) Triton X-100 in PBS to minimize nonspecific binding.

Primary antibodies against EEA1 and RAB7 were diluted in PBS supplemented with CaCl₂ and MgCl₂, containing 1% (v/v) Tween^®^ 80, and added to the wells (100 µL per well). Cells were incubated with primary antibodies for 24 h at 4 °C. The following day, cells were washed five times with PBS (5 min per wash).

Secondary antibodies (goat anti-mouse Alexa Fluor^®^ 647 and goat anti-rabbit Alexa Fluor^®^ 488) were diluted in the same buffer as the primary antibodies, dispensed into the wells, and incubated for 45 min at RT. Cells were then washed five times with PBS (5 min per wash). Lastly, nuclei were stained with Hoechst (1:5000 dilution in PBS; 100 µL per well). Cells were imaged immediately using a BioTek Cytation C10 confocal imaging system (Agilent Technologies, Inc.).

### Biological evaluation in a 3D MDA-MB-231 model

Spheroids were generated by seeding MDA-MB-231 cells into Costar Corning^®^ 96-well round-bottom microplates using DMEM supplemented with 10% FBS, 2 mM L-glutamine, and 50 U/mL penicillin–streptomycin. To optimize spheroid formation, cell seeding densities ranging from 2 × 10^3^ to 4 × 10^4^ cells per well were evaluated. The optimal condition for uniform spheroid formation was determined to be 2 × 10^3^ cells per well. Cells were incubated for 72 h, and spheroid formation was monitored by optical microscopy.

Spheroids were subsequently treated with fluorescently labeled siRNA_SC_-loaded dcNP2.0 dispersed in DMEM supplemented with 1% FBS. dcNP2.0 internalization within spheroids was evaluated using the same procedure described above for 2D cultures and monitored using an Incucyte^®^ SX5 live-cell imaging system.

To investigate the role of CD44 in dcNP2.0 uptake, a competitive inhibition assay was performed by pretreating spheroids with free HA (10 mg/mL) for 4 h. Following pretreatment, the medium was gently removed and replaced with fresh medium containing siRNA_SC_-loaded dcNPs2.0 prepared using the PLGA/PLGA–Rhodamine mixture. dcNP2.0 internalization was monitored as described above using the Incucyte^®^ SX5.

For confocal imaging of dcNP2.0 penetration into the spheroid core, spheroids were fixed at the end of the experiment (72 h) with 4% (w/v) PFA, washed with PBS, stained with Hoechst (1:5000 dilution in PBS) for 30 min, mounted using ProLong^®^ Gold antifade mounting, and covered with coverslips. Spheroids were imaged using a BioTek Cytation C10 Confocal Imaging Reader (Agilent Technologies, Inc.) equipped with a 20 × confocal objective.

For gene silencing studies, spheroids of MDA-MB-231 cells stably expressing luciferase were treated with siRNA_LUC_-loaded dcNPs2.0 for 72 h, after which luciferase expression was quantified as described previously. In parallel, spheroids of MDA-MB-231 cells stably expressing GFP were prepared using the same procedure and treated with siRNA_GFP_-loaded dcNPs2.0. GFP silencing was monitored over 72 h by measuring green fluorescence using the Incucyte^®^ SX5 system. Lipofectamine was used as a positive control.

### Statistical analysis

All data were analyzed by GraphPad Prism 9. All the values were presented as mean ± SD. Comparison among the different groups was performed by one-way ANOVA and two-way ANOVA.

## Supplementary Information

Below is the link to the electronic supplementary material.

ESM 1 (DOCX 413 KB)

## Data Availability

The data that support the findings of this study are available from the corresponding author upon reasonable request.
