# Supplementary material for: Double-coated PLGA nanoparticles with hierarchical surface architecture for CD44-targeted siRNA delivery
Source: Drug Deliv Transl Res. 2026 May 8;16(10):3956–71. doi: 10.1007/s13346-026-02115-8 (PMC13619804; doi:10.1007/s13346-026-02115-8)
Supplement: Supplementary file 1 [file 13346_2026_2115_MOESM1_ESM.docx]

Supporting Information

Double-Coated PLGA Nanoparticles with Hierarchical Surface Architecture for CD44-Targeted siRNA Delivery

*Giuseppe Longobardi^1,2^, Pini Shekhter^3^, Claudia Conte^1^, Ronit Satchi-Fainaro^2,3,4*^, Fabiana Quaglia^1*^*

^1^Department of Pharmacy, University of Naples Federico II, Naples 80131, Italy

^2^Department of Cancer Biology & Immunology, Gray Faculty of Medical and Health Sciences, Gray School of Medical Sciences, Tel Aviv University, Tel Aviv 6997801, Israel.

^3^Tel Aviv University Center for Nanoscience and Nanotechnology, Tel Aviv University, Tel Aviv 6997801, Israel.

^4^Sagol School of Neuroscience, Tel Aviv University, Tel Aviv 6997801, Israel.

**Table S1**	S2

**Figure S1**	S2

[**Figure S2**	S3](#_Toc194676006)

[**Figure S3**	S3](#_Toc194676006)

**Table S2**	S3

**Table S1**. Stepwise colloidal properties of dcNPs2.0 during formulation and surface layering.

|  | **D_H_ (nm ± SD)** | | **PDI (± SD)** | | **ζ (mV ± SD)** | **Yield (%)** | |  |
| --- | --- | --- | --- | --- | --- | --- | --- | --- |
| dcNPs2.0@PLGA | 118 ± 2 | 0.05 ± 0 | | -36 ± 2 | | |  | |
| dcNPs2.0@PEI | 125 ± 1 | 0.05 ± 0 | | +62 ± 2 | | |  | |
| dcNPs2.0@PEI_siRNA_ | 135 ± 2 | 0.04 ± 0 | | +55 ± 2 | | |  | |
| dcNPs2.0@HA_siRNA_ | 158 ± 2 | 0.05 ± 0 | | -30 ± 1 | | | 99 ± 1 | |
| [dcNPs2.0@HA_siRNA_](mailto:dcNPs2.0@HAsiRNA) filtered 0.45μm | 147 ± 2 | 0.04 ± 0 | | -35 ± 1 | | | 80 ± 4 | |

**Figure S1.** Reference XPS spectra used for depth-profile analysis shown in the main text. A) High-resolution C 1s spectrum showing deconvolution of the main carbon chemical states associated with the dcNP2.0 core. B) High-resolution N 1s spectrum corresponding to nitrogen-containing species, primarily attributed to the PEI interlayer. C) High-resolution P 2p spectrum used as a reference for siRNA, confirming the presence of phosphate groups characteristic of the nucleic acid backbone.

**Figure S2.** CD44 expression assessed by flow cytometry. Representative fluorescence histogram (CD44–PerCP) of cells stained with anti-CD44 antibody (red) overlaid with isotype control (gray) and unstained (black) samples. The rightward shift of the stained population relative to controls indicates robust CD44 surface expression.

**Figure S3.** Uptake of dcNPs2.0-siRNA_SC_ in 2D TNBC models. Representative fluorescence images of spheroids after 72 h incubation with dcNPs2.0-siRNA_SC_ at 0.13 mg/mL (top) and 0.052 mg/mL (bottom). Scale bar: 400 µm.

| **Table S2.** Composition and properties of dcNPs2.0 prepared using the mixture of PLGA/PLGA-Rhodamine. | | | | |
| --- | --- | --- | --- | --- |
|  | D_H_  (nm ± SD) | PDI  (± SD) | ζ  (mV ± SD) | |
| dcNPs2.0-siRNA_SC_ filtr 0.45 μm | 144 ± 5 | 0.05 ± 0 | | -25 ± 1 |
